# Construction of land-use change matrix and estimation of greenhouse gas inventory focusing on settlements in South Korea

**DOI:** 10.1186/s13021-023-00223-3

**Published:** 2023-03-21

**Authors:** Sol-E Choi, Segi Hong, Cholho Song, Jiwon Kim, Whijin Kim, Ram Ha, Woo-Kyun Lee

**Affiliations:** 1 Department of Environmental Science and Ecological Engineering, Korea University, Seoul, 02841 Republic of Korea; 2 OJEong Resilience Institute (OJERI), Korea University, Seoul, Republic of Korea

**Keywords:** Land use, Land use change, Settlements, Greenhouse gas inventory, Land-use change matrix

## Abstract

**Background:**

Five ministries are involved in estimating the greenhouse gas (GHG) inventory in the South Korean land use, land-use change, and forestry (LULUCF) sectors. However, these ministries have not established a consistent land classification standard between land-use categories. Therefore, the GHG inventory is estimated at the approach 1 level with no spatial clarity between land-use categories. Moreover, the settlements category is not estimated because activity data and the spatial scope are lacking. This study proposed a methodology for constructing a land-use change (LUC) matrix in the LULUCF sector for improving approach level and estimating the GHG inventory in the settlements.

**Result:**

We examined 10 sets of spatiotemporal data in South Korea to construct a LUC matrix. To maintain consistency in the spatial land classification, we constructed a LUC matrix using cadastral maps, which provide useful data for consistent land-use classification in South Korea. The LUC matrix was divided into remaining and land-converted settlements between 2005 and 2019 with estimated areas of 878,393.17 and 203,260.42 ha, respectively. CO_2_ emissions, according to Intergovernmental Panel Climate Change’s Guideline Tier 1, were estimated at 18.94 MtCO_2_ for 15 years, with an annual CO_2_ emission of 1.26 MtCO_2_ yr^−1^. CO_2_ emission by land conversion type was found to be the largest at 16.93 MtCO_2_ in the case of forest converted to settlements. In addition, the area with the largest CO_2_ emission density was Sejong-si at 7.59 tCO_2_/ha.

**Conclusion:**

Based on reviewing available spatial data in South Korea, it is possible to improve Approach 3, which is more advanced than previous Approach 1 in the settlement category. In addition, the national GHG inventory also can be estimated by our constructed LUC matrix and activity data in this study. Under the many discussions about developing the Approach system, this study can provide in-detail information on developing LUC in South Korea in the settlement category as well as suggesting a methodology for constructing the LUC matrix for countries with similar problems to South Korea.

## Background

Estimation of greenhouse gas (GHG) emissions from anthropogenic sources and removals by sinks is an important contribution of the Parties to the United Nations Framework Convention on Climate Change (UNFCCC) towards meeting their commitments under the convention [1]. By adopting the Kyoto Protocol in 1997, the international communities, particularly the Annex countries, were obligated to reduce GHGs and report their national inventories. As part of the Paris Agreement (PA), all parties agreed to set and achieve their own GHG reduction goals (Nationally Determined Contributions). In addition, the parties agreed that Nationally Determined Contributions should be developed gradually (Article 4.3, PA [2]) and that progress towards the agreement's goals should be tracked, with the pace of progress evaluated during 5‐yearly Global Stocktakes (Article 14, PA [2]). The mitigation objectives declared in the PA would require the parties to reach and sustain net zero global anthropogenic CO_2_ emissions between 2050 and 2075. Land use, land-use change, and forestry (LULUCF) sectors play important roles in the climate system as both a source and sink of GHGs and by providing contributions to the goals of the UNFCCC. Most parties have acknowledged that the LULUCF sector may have substantial implications for the ambition level of their target [3]. Therefore, carbon removals/emissions from LULUCF have received increased attention [4]. Accordingly, calculation of the GHG inventory is essential for the LULUCF sectors.

The Intergovernmental Panel on Climate Change (IPCC) provides guidelines on a reporting-monitoring-verification method for GHG inventories. The Good Practice Guidance for LULUCF (GPG-LULUCF) [5] not only supports the development of inventories, but also demands the classification of the LULUCF sector (forest land, cropland, grassland, wetlands, settlements, and other land). To comply with the GPG–LULUCF, inventory agencies at the national level need information about land area for each of six categories to estimate carbon stocks and emissions and removals of GHG associated with LULUCF activities [6]. The LULUCF sector’s GHG inventory requires construction of a land-use change (LUC) matrix that divides the remaining in the same category and those converted from other categories to estimate the GHG inventory accurately [7]. Article 4.13 of the PA requests the promotion of transparency, accuracy, completeness, comparability, and consistency (TACCC) and prevention of double accounting. The importance of constructing a LUC matrix in the LULUCF sector is being emphasized in recent works. IPCC provided three generic approaches (App) for land-use identification for estimating the precise area. Tiers 1–3 can be determined based on the level of the App or development of the country-specific removal/emission factors [8]. In the LULUCF sector, high-level App and Tiers explain land use and changes in more detail. Therefore, high-level GHG inventory requires consistent standards for land-use classification and land-use change within the LULUCF sectors. For example, in countries such as the United States, Australia, the Netherlands, and New Zealand, the central management office prepares the GHG inventory report for the LULUCF sector by integrating and coordinating spatial data and land-use classification. These countries accumulate the time series of spatial data for inventory reports using App 3 through a unified land-use classification [9–12]. In Italy, App 2 is applied to identify the land-use area based on a sampling survey of the National Land-Use Inventory fixed sample site [13]. Japan calculates the area using App 2, which determines spatial boundaries according to hierarchical rankings, using data from Land Use Status Survey, National Forest Resource Databases and Cultivated and Planted Area Statistics [14].

The government of South Korea enacted the ‘Act on Low Carbon Green Growth’ for comprehensive GHG management regulation in 2010 and began systematically obtaining statistics for five sectors: energy, industrial, agriculture, waste, and LULUCF [7]. Although the unified management of GHG improved the overall precision and accuracy for estimating GHG inventory, the LULUCF sector has difficulty improving App level because of using different reporting and estimation ministries systems for the land-use category. The difficulty in improving App level is also related to the social and economic situation in South Korea. Because of rapid land and economic development in the 1960s in South Korea, the ministries in the LULUCF sector often constructed various national statistical data (cadastral statistics, forest basic statistics, agricultural area survey) and national spatial data (National Forest Inventory data, cadastral map, smart farm map, land cover map) to provide basic data for the government’s policies. In South Korea's land management, the Ministry of Land, Infrastructure and Transport (MOLIT) establishes and manages the space plan for the land. However, since detailed management of each land-use category is conducted by the Ministry of Agriculture, Food and Rural Affairs (MAFRA), the Ministry of Oceans and Fisheries (MOF), there is a lack of consistency in spatial classification according to the management scope. Therefore, the GHG inventory has been reported without integrating land-use classification in the LULUCF sector, leading to an App 1 level of estimation. The total area and land-use categories that changed were not distinguishable, and statistics on land-use conversions are not available [7]. Accordingly, the LULUCF sector currently estimates GHG inventory from the total area derived according to the definition of each category as the land area maintained as the same land without information from LUC. Moreover, the settlements category has not been reported because the spatial boundaries have not been determined and data on GHG removals and emission activity are lacking. Therefore, these data must be integrated to improve GHG inventory management.

According to the Ministry of Statistics in Korea (KOSTAT), the area of cropland decreased from 1,898,000 ha in 2000 to 1,565,000 ha in 2019 [15] and that of forest land decreased from 6,422,128 to 6,299,276 ha [16]. Settlements are expanding because of continuous development, however, these statistical data can not provide detailed information on land conversion. Thus, spatial boundaries should be clearly determined in all categories of the LULUCF, including in settlements. A LUC matrix must be constructed, and GHG inventory estimation and land management plans should be established. Additionally, to ensure TACCC in the LULUCF sector, the GHG inventory should be estimated based on activity data reflecting the LUC at App 3. This study was conducted to construct the LUC matrix and activity data in the LULUCF sector to improve the level of Tier and App in other countries with similar conditions as South Korea. We first reviewed the available spatial data to set spatial boundaries in the LULUCF sector. Next, we constructed the LUC matrix and activity data focused on settlements to calculate the GHG inventory. Finally, we estimated the GHG inventory and discussed the local and international applicability of the measurement, reporting, and verification (MRV) guidelines.

### Study area

**Fig. 1:**
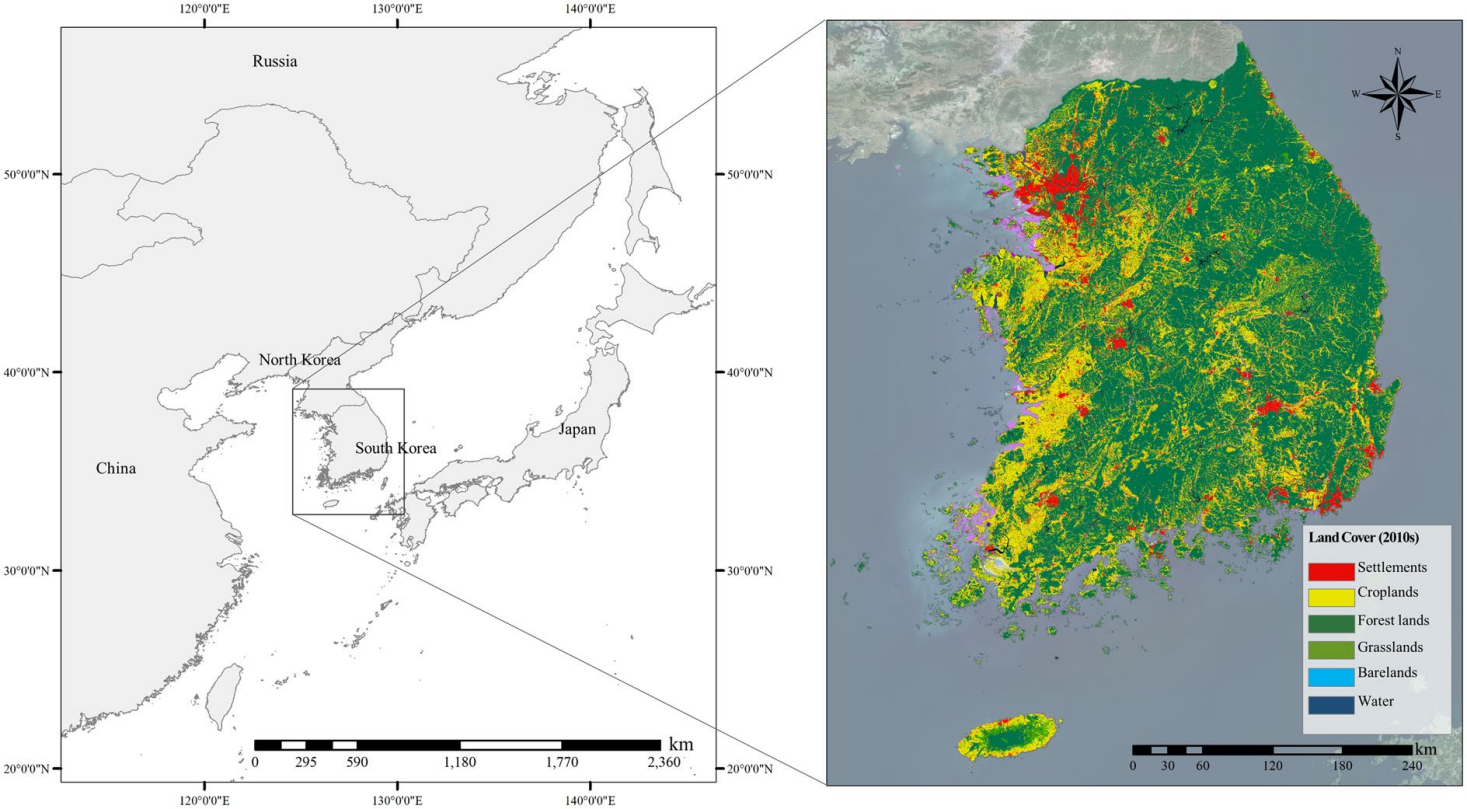
Location and land cover map of South Korea in the 2010s

This study focused on South Korea (37° N latitude and 127°  E longitude), which occupies a total area of 100,412 km^2^ and is geographically located in East Asia (Fig. 1). Land use statistics from 2010 based on cadastral statistics showed that forest lands make up 64,471.9 km^2^, croplands make up 19,715.6 km^2^, and settlements and other lands make up 15,709.5 km^2^ in South Korea. In 2020, forest land accounted for 63,558.3 km^2^, cropland for 18,654.4 km^2^, and settlements and other lands for 18,199.9 km^2^; therefore, forest land and cropland are gradually decreasing, whereas settlements are expanding [17, 18]. As of 2020, the number of people living in urban areas was 47.57 million, accounting for 91.8% of the population. In South Korea, continuous development has occurred under the guidance of policies for creating new cities to enable population density control and urban distribution, leading to LUC. Moreover, South Korea recently shifted from a developing country to a developed country, and reductions in GHGs will likely be demanded by the international community. South Korea has established specific policies and plans for managing the GHG inventory in accordance with laws and regulations such as ‘The Act on Low Carbon Green Growth’ and ‘Regulations on the General Management of National Greenhouse Gas Statistics’. However, the LULUCF sector is estimated to have a low Tier and App because of the overlapping scope of management targets among ministries. Furthermore, the GHG inventory system and land management system are not unified. Therefore, the LULUCF GHG inventory cannot reflect the current status of land use and land-use changes. South Korea is a suitable study area for improving methods used for GHG inventory in the LULUCF sector while considering the needs of the international community and specific national conditions.

## Methods

**Fig. 2:**
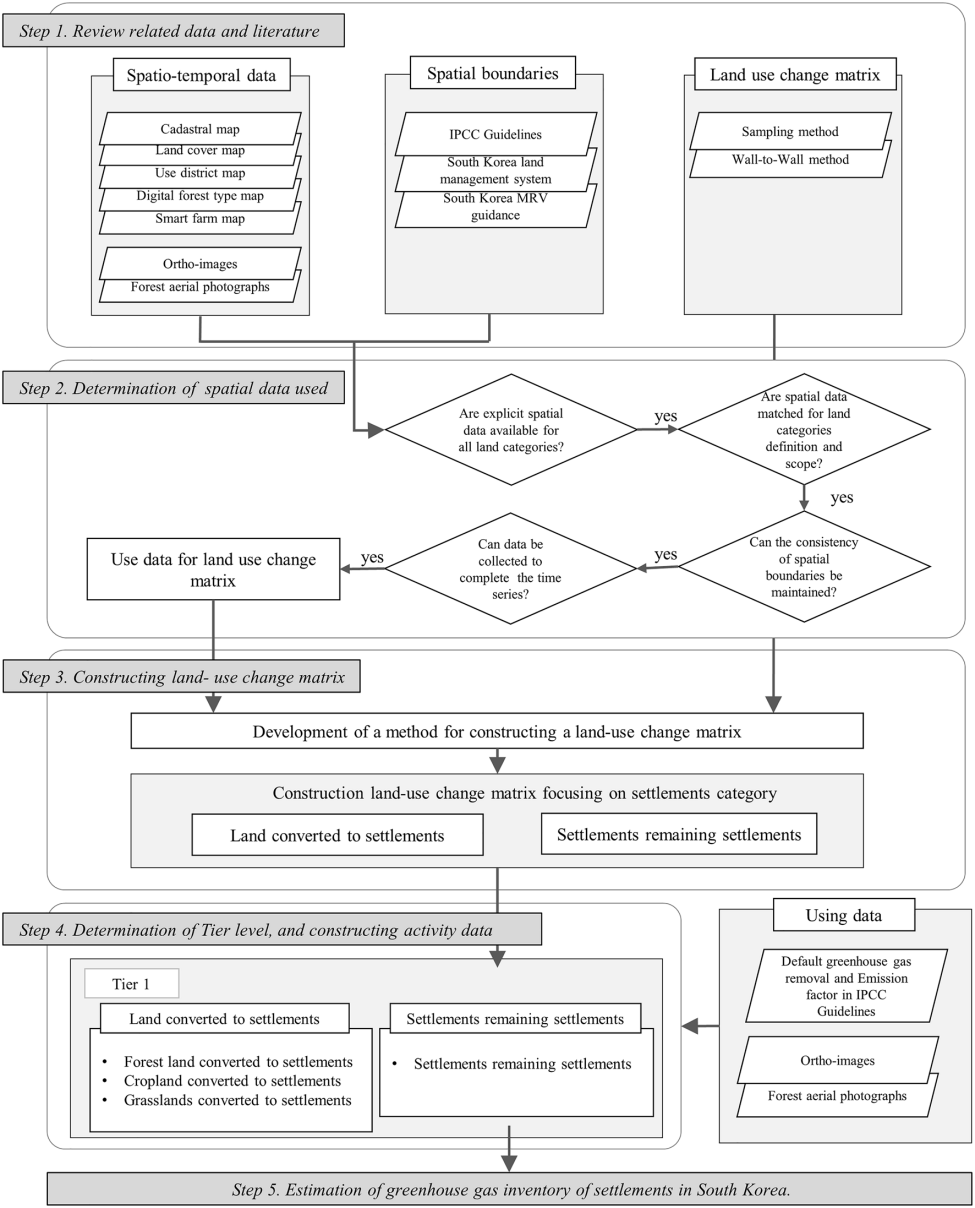
Overall study flow of the study

This study involved construction of a LUC matrix and GHG inventory statistics focusing on the settlements category in five steps (Fig. 2). In Step 1, we reviewed spatial data, defined the spatial boundary, and constructed a method for generating a LUC matrix. Based on this information, each land use category’s spatial boundary was determined and selected corresponding to the spatial data in Step 2. In Step 3, the LUC matrix was constructed to include land converted to settlements (LS) and settlements remaining as settlements (SS). In Step 4, activity data according to the LUC matrix were constructed. In Step 5, the GHG inventory statistics of the settlements were estimated using the activity data and GHG removal/emission factors.

### Step 1: Review related spatial data and literature

In South Korea, ministries such as the Ministry of Land, Infrastructure and Transport, Korea Forest Service, and Ministry of Agriculture, Food and Rural Affairs are included in the LULUCF sector. Each ministry constructs and manages spatial data according to the object and purpose of management but at different spatiotemporal resolutions. Therefore, we reviewed spatial data for the LUC matrix and spatial data that could be used to estimate the GHG inventory of the settlements category. Additionally, the spatial boundaries and method for constructing the LUC matrix were reviewed in the Good Practice Guidance 2003-LULUCF, IPCC 2006 GL, South Korea's land management system, and MRV guidance.

### Step 2: Determination of spatial data used

The spatial data used to construct the LUC matrix was determined by reviewing four main factors. First, we considered whether spatial data are explicit with all land-use categories. Second, whether spatial data matched the definition of each land-use category. Third, it was considered whether data maintain the consistency of spatial boundaries, and fourth, whether spatial information can be collected in time series.

### Step 3: Constructing land-use change matrix

IPCC guides three generic methods for representing land areas. App 1 identifies the total area for each land-use category within a country but does not provide detailed information on land-use conversion. App 2 introduces information for tracking quantitative changes in each land-use category. App 3 extends the information available in App 2 by allowing land-use conversion to be tracked on a spatially explicit basis [19]. To construct the LUC matrix of App 3 guided by Good Practice Guidance for LULUCF, the sampling method, and wall-to-wall method were analyzed and used for the South Korea situation.

### Step 4: Determination of tier level and construction activity data

IPCC guides Tier 1–3 depending on the development situation of country-specific removals and emission factors. Tier 1 applies the default factors provided in the IPCC Guidelines (GL). Tier 2 applies country-specific factors developed for biomass, soil, and dead organic matter. At Tier 3, higher-order methods are used, including complex models and repeated inventory measurement systems. We applied Tier 1 for biomass because South Korea’s country-specific removal and emission factors are not developed in the LULUCF sector except in forest lands. The Tier 1 method assumes no change in the carbon stock in live biomass in SS and only estimates the LS. For the LS, during the initial year following conversion to settlements, the most conservative approach is to set the biomass to zero after conversion, which means that the development of settlements completely depletes the carbon stocks [8]. We also used default factors provided for each land use category in the 2006 IPCC GL to estimate carbon dioxide removals before conversion (Table 1). Among the calculation items (biomass, litter, dead wood, soil carbon), a biomass map that can be constructed using remote sensing data was constructed and used as activity data. Activity data were constructed by visual interpretation of forest aerial photographs (0.8 m resolution) in Esri's ArcGIS program.

**Table 1:** Land conversion default removal and emission factors for Tier 1 in Intergovernmental Panel on Climate Change Guidelines

Land-use category	Carbon stock in biomass before conversion	
Forest land	Temperate continental forest	120 t d.m.ha^−1^
Cropland	For cropland containing annual crops	4.7 C ha^−1^
Grassland	Warm temperate-wet	13.5 t d.m.ha^−1^

d.m: dry matter (basic wood density)

### Step 5: Estimation of GHG inventory and uncertainty

Calculation of greenhouse gas inventories of settlements is based on the activity data that has been constructed, divided into forest land converted to settlements (FS), cropland converted to settlements (CS), and grassland to settlements (GS). Emissions were estimated according to each land conversion type. In addition, we calculated and comparison the carbon emission density associated with the conversion of each county.

Accounting for GHG inventory requires an assessment of uncertainty. LULUCF is a large source of uncertainty in estimating anthropogenic GHG emissions [20–23]. The uncertainty assessment defined by the IPCC is a method of quantifying and expressing the degree of uncertainty in emissions and removals estimated in national GHG inventories. Uncertainty is, therefore, a quantitative measure of the reliability of GHG emissions. The GHG inventory utilizes a method of determining each variable’s uncertainty and summing each element's uncertainty into the overall inventory uncertainty. In general, the estimation of GHG inventory uncertainty can be based on measurement data, domestic and international literature, IPCC default uncertainties, or expert judgment [24]. In this study, the uncertainty of the coefficients was not calculated because the default coefficient values presented at the IPCC were used. In the uncertainty of activity data, we examined literature and other data that can be compared and performed a comparative analysis of GHG emissions.

## Materials

The spatial data and remote sensing data used for the review and construction of the LUC matrix were downloaded from the website managed by each ministry (Table 2).

**Table 2:** Data used for review in this study

Data name	Source of reviewed data	Management ministry
Cadastral map	National Spatial Infrastructure Portal (http://data.nsdi.go.kr/dataset)	MOLIT
Use district map	National Spatial Infrastructure Portal (http://data.nsdi.go.kr/dataset)	MOLIT
Land cover map	Environmental Geographic information system (https://egis.me.go.kr/atlas/list.do)	ME
Digital forest type map	Forest Geospatial Information System (https://fgis.forest.go.kr/)	KFS
Smart farm map	Agricultural and Rural Affairs Farmap Service (https://agis.epis.or.kr/)	MAFRA
Ortho-images	National Geographic Information Institute (https://www.ngii.go.kr/kor/)	MLIT
Forest aerial photographs	Forest Geospatial Information System (https://fgis.forest.go.kr/)	KFS

KFS, Korea Forest Service; MAFRA, Ministry of Agriculture, Food and Rural Affairs; MOLIT, Ministry of Land, Infrastructure and Transport

To determine the spatial boundaries, we also reviewed the IPCC definition, spatial definition of South Korea’s land management system, and GHG inventory MRV Guidance (Table 3).

**Table 3:** Comparison of land use, land-use change, and forestry sector definition between Intergovernmental Panel on Climate Change Guidelines (IPCC GL), South Korea land management system and Measurement, Reporting, and Verification (MRV) Guidance

Division	IPCC GL [8]	Land management system [25]	Current MRV guidance
Forest land	All land with woody vegetation consistent with thresholds used to define forest land; also includes systems with a vegetation structure that currently falls below, but in situ could reach, the threshold values used	Land spanning more than 0.5 ha with trees higher than 5 m and a canopy cover of more than 10% and minimum width of more than 30 m	Coniferous, deciduous, mixed forests and bamboo areas of basic forest statistics
Cropland	Cropped land, including rice fields, and agroforestry systems in which the vegetation structure falls below the thresholds used for forest land	Cropped land including rice fields and orchards	Paddy, field, and orchard of agricultural area survey
Grassland	Rangelands and pastureland are not considered as cropland; also include systems with woody vegetation and other non-grass vegetation	Rangelands and pastureland considered as cropland	Pastureland of cadastral statistics
Wetlands	Peat extraction and land covered or saturated by water for all or part of the year and that does not fall into other categories	Areas of peat extractions and land covered or saturated by water for all or part of the year	Rivers, drains, reservoirs, and fish farms of cadastral statistics
Settlements	All developed land, including transportation infrastructure and human settlements of any size, unless they are already included in other categories	All developed land	Nineteen categories of cadastral statistics
Other lands	Bare soil, rock, ice, and all land areas that do not fall into any of the other five categories	Undefined	Undefined

## Results

### Review related spatial data and literature

The spatial data for determining the spatial boundaries and constructing the LUC matrix were analyzed by dividing the data based on the content, time series, and spatial data types (Table 4). The cadastral map used in the study consisted of 28 categories according to South Korea’s cadastral status, including forests, fields, paddies, and roads. It also functioned as a basic topographic map with necessary data for urban planning and farmland management. Generally, cadastral map shows the boundaries and ownership of land parcels that separate adjacent land plots and contains both spatial information (shape, area, boundary, and location) and non-spatial information (land use, value, and tenure) encoded in the attribute information [26]. Therefore, it is useful for estimating the spatial area of changes in detailed land categories when estimating the GHG inventory. However, because the GHG removal and emission source information is not included in the map, it is necessary to construct activity data for estimating the biomass change in land use for 20 years presented by the IPCC. The use district map shows spatial data that divides the land into urban areas, management areas, agricultural and forestry areas, and natural environment conservation areas, and includes land use regulations for land management. The spatial boundaries of forest land, cropland, and settlements and information on land use plans can be determined; however, it is difficult to estimate the current land use status in detail because the map has set the content and range of regulations for land use planning and management. The Digital Forest Type map shows the forest distribution in South Korea and includes the forest type, species, and age for estimating the GHG inventory [27]. But does not contain information on other categories. The land cover map shows the current state of the ground surface determined by analyzing remote sensing images [28]. However, it is difficult to estimate the land management status according to land use because information on only the land cover state is provided. A smart farm map is a digital map of cropland based on high-resolution aerial images of the spatial area but contains no data on the past. Therefore, the spatial boundary of each category was determined using cadastral map to ensure that the spatial boundary was consistent with other categories.

**Table 4:** Spatial data review for constructing land use, land-use change matrix, and activity data

Data name	Description	Time series coverage	Spatial resolution and data type
Cadastral map	Map prepared by dividing the national land into 28 categories according to land-use purpose and status	1970s–present (renewed monthly)	Vector
Use district map	Areas determined by urban management plans do not overlap with economical and efficient land use and promote public welfare by limiting land use	2005–present (renewed monthly)	Vector
Ortho-images	Data produced through orthometric correction using aerial photographs of South Korea	2002–present (renewed every 2 years)	Raster with 12 cm (urban area), 25 cm (others)
Digital forest type map (1:25,000 scale)	Map of forest information on National Forest Resource Survey	1st (1971–1974) 2nd (1978–1980) 3rd (1986–1992) 4th (1996–2005) 5th (2006–2010)	Vector
Digital forest type map (1:5000 scale)	Map of detailed forest information using digital aerial photographs, ortho-images, and 1:25,000 Forest Type Map	2009–2013	Vector
Forest aerial photographs	Black-and-white aerial images constructed over 4 periods in South Korean territory	1st (1971–1974) 2nd (1978–1980) 3rd (1986–1992) 4th (1996–2005)	Raster with 0.8 m
Land cover map (1:50,000 scale)	Spatial data represented by classifying land cover types into seven categories	Construction (1998), Advanced (2000), Actualizing (2010), Update (2019)	Vector
Land cover map (1:25,000 scale)	Spatial data represented by classifying land cover types into 22 categories	Construction (2004) 1st update (2007) 2nd update (2009) 3rd update (2013) update (2018)	Vector
Land cover map (1:5000 scale)	Spatial data represented by classifying land cover types into 41 categories	Constructed by region (2010–2016)	Vector
Smart farm map	Map of agricultural land constructed using high-resolution aerial images	2014–2018	Vector

### Use of spatial data

The spatial boundaries were set using available cadastral maps while considering the definition, consistency, and time series between land-use categories. Specific categories in the cadastral map were classified according to those in the LULUCF (Table 5). Settlements were determined to be comprised of 19 categories, not including ‘other’ categories.

**Table 5:** The spatial boundary of land use, land-use change, and forestry (LULUCF) sector using a cadastral map in South Korea

Categories in LULUCF	Categories in cadastral map
Forest land	Forest land
Cropland	Field paddy field, orchard
Grassland	Ranch
Wetlands	River, ditch, reservoir, fish farm
Settlements	Mineral spring site, salt farm, site, factory site, school site, parking zone, gas station, storage site, road, railroad, embankment, waterways, park, physical site, amusement park, religion site, historic site, graveyard, miscellaneous land
Other land	–

### Constructing land-use change matrix and activity data

**Fig. 3:**
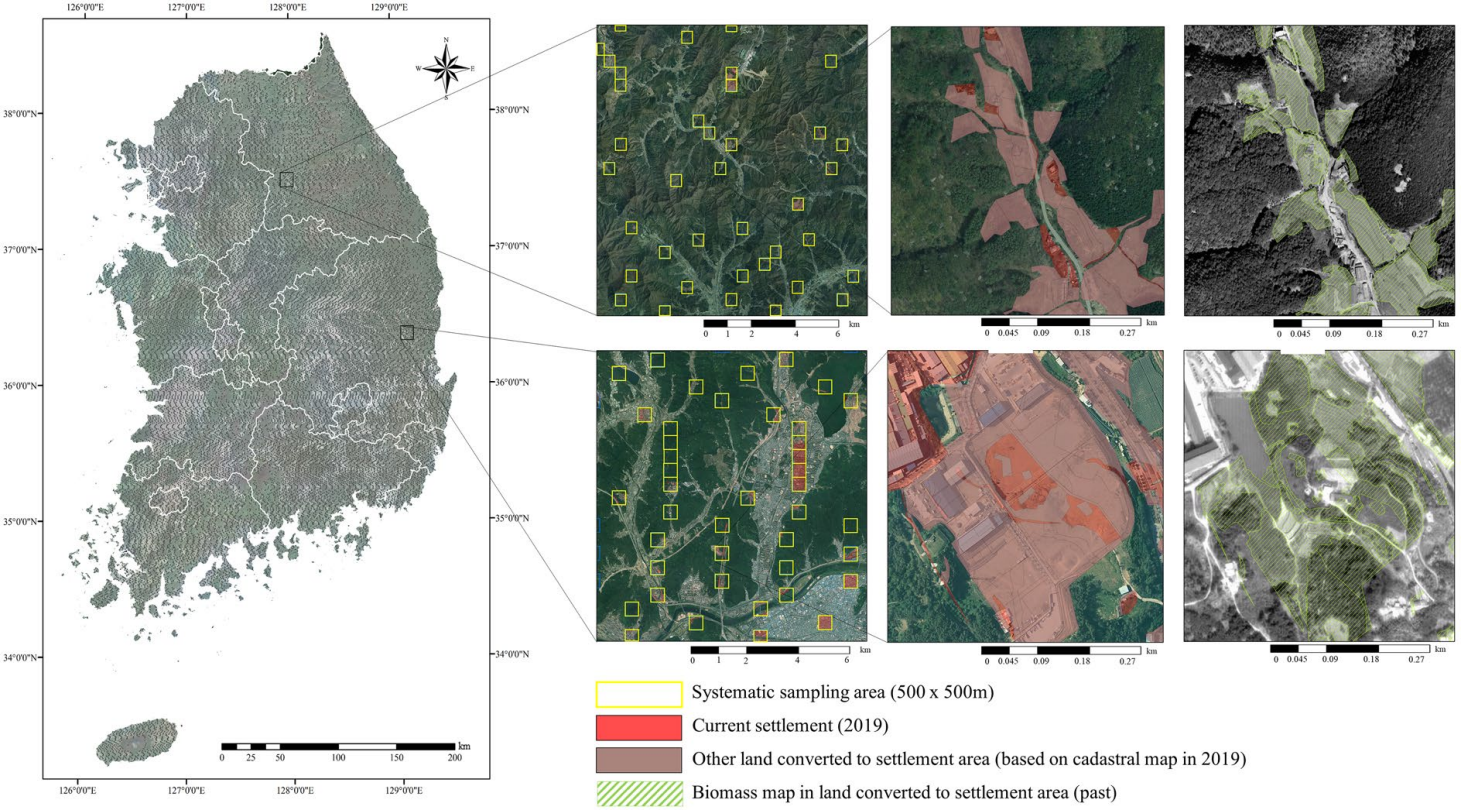
Sampling areas and construction method of land-use change matrix and activity data. **a** distribution of sampling area, **b** The spatial extent of the settlements defined by the cadastral map, and the spatial range of settlements remaining settlements, and land converted to settlements. **c** activity data (biomass map) conducted by visual interpretation

Of the 37 Annex I parties, 15 constructed matrices using sampling methods, 14 used both sampling and wall-to-wall methods, and the others used only the total land area of national statistical data [29]. In the wall-to-wall method, a theme map is constructed for the LUC using remote-sensing data based on the time series of a theme map or combination with other data. The sampling method directly estimates land use and land-use changes through the repetitive sampling of different areas obtained through field surveys or remote sensing data. In South Korea, Park et al. [30, 31] and Yu et al. [7] attempted to construct a LUC matrix using remote sensing data based on sample points using a random sampling method. Park et al. [32] analyzed the advantages and disadvantages of applying the sampling and wall-to-wall methods to forest lands in South Korea. As the sampling method detects land-use changes based on the sample points, the time-series conversion can be identified easily, whereas it is difficult to determine the boundaries of each category. In contrast, the wall-to-wall method reveals differences in the spatial boundaries between land cover and land use. By combining the results of these prior studies, the sample area was extracted using the cadastral map as primary data, and activity data were obtained by combining the wall-to-wall and sampling methods to construct a LUC matrix for the whole sample area. To construct a LUC matrix according to the land-use status between 2005 and 2019, particularly settlements, the 32,071 sampling area (grid) was extracted using a systematic sampling method by sampling areas from 39,390 grids, which is 10% of the grid South Korea’s land into 500 m × 500 m (Sample size, a greater than 35,003 derived from a population of 393,898 with a 95% confidence level and a sampling error of 5%, was used) (Fig. 3).

The sampling ratio for each province was calculated to estimate the total land-converted area using the data derived from the sample area (Table 6).

**Table 6:** Ratio of sampling area to total current settlements areas

Division	Total settlements area (ha)	Settlements in sampling area (ha)	Sampling ratio (%)
Gangwon-do	78,072.89	7463.95	10.46
Gyeonggi-do	201,263.03	24,786.09	8.12
Gyeongsangnam-do	103,032.89	9692.65	10.63
Gyeongsangbuk-do	118,482.20	11,812.78	10.03
Gwangju-si	15,932.51	1625.77	9.80
Daegu-si	23,378.41	2289.76	10.21
Daejeon-si	15,296.09	1514.46	10.10
Busan-si	27,748.82	2627.73	10.56
Seoul-si	38,659.02	3920.79	9.86
Sejong-si	7598.85	762.17	9.97
Ulsan-si	19,574.74	1880.38	10.41
Incheon-si	37,359.91	3139.49	11.90
Jeollanam-do	121,483.69	11,023.93	11.02
Jeollabuk-do	80,440.75	7832.59	10.27
Chungcheongnam-do	97,995.02	9,597.95	10.21
Chungcheongbuk-do	68,026.56	6,843.72	9.94
Jeju-si	27,308.21	2,568.98	10.63
Total	1,081,653.59	105,630.23	10.24

We constructed a settlement LUC matrix such as FS, CS, and GS. For wetlands, it appeared that no areas were converted to settlements during the study period, thus, these data were excluded from analysis. The SS and LS were 878,393.17 ha (81.21%) and 203,260.42 ha (18.79%), respectively, at the national level. Gyeonggi-do is the largest SS area (19,927.00 ha), followed by Jeollanam-do (8,914.11 ha), Gyeongsangbuk-do (8852.8 ha), Gyeongsangnam-do (8264.92 ha), and Chungcheongnam-do (7419.17 ha). Seoul-si (97.37%) showed the highest percentage of SS, followed by Incheon-si (92.06%), Jeollabuk-do (87.17%), Chungcheongbuk-do (86.61%), and Gyeongsangnam-do (85.27%) (Table 7).

**Table 7:** Settlements remaining settlements in South Korea

Unit: ha (%)			
Division	Settlements remaining settlements		
	Sampling area	Total area	Ratio
Gangwon-do	5857.72	61,268.73	78.48
Gyeonggi-do	19,927.00	161,803.71	80.39
Gyeongsangnam-do	8264.92	87,860.59	85.27
Gyeongsangbuk-do	8852.80	88,792.70	74.94
Gwangju-si	1224.92	12,005.86	75.35
Daegu-si	1860.79	18,997.21	81.26
Daejeon-si	1273.90	12,866.10	84.11
Busan-si	2117.94	22,363.92	80.59
Seoul-si	3817.61	37,641.90	97.37
Sejong-si	441.73	4404.42	57.96
Ulsan-si	1370.07	14,262.17	72.86
Incheon-si	2890.37	34,395.03	92.06
Jeollanam-do	8914.11	98,238.14	80.87
Jeollabuk-do	6828.44	70,123.75	87.17
Chungcheongnam-do	7419.17	75,751.69	77.30
Chungcheongbuk-do	5926.87	58,916.68	86.61
Jeju-si	1758.85	18,700.58	68.48
Total	84,994.26	878,393.17	81.21

Gyeonggi-do is the largest LS area (39,459.32 ha), followed by Gyeongsangbuk-do (29,689.50 ha), Jeollanam-do (23,245.55 ha), Chungcheongnam-do (22,243.33 ha), and Gangwon-do (16,804.16 ha). Sejong-si (42.04%) showed the highest percentage of LS (%), followed by Jeju-si (31.52%), Ulsan-si (27.14%), Gyeongsangbuk-do (25.06%), Chungcheongnam-do (22.70%), and Gyeonggi-do (19.61%). The national average estimated 18.79% of the LS. In addition, according to the LULUCF sector, CS (84,401.37 ha, 7.80%) was estimated to account for the largest area, followed by FS (74,502.57 ha, 6.89%) and GS (44,356.48 ha, 4.10%) (Table 8).

**Table 8:** Land converted to settlement areas in South Korea

Unit: ha (%)								
Division	Land converted to settlements (A + B + C)		Forest converted to settlements (A)		Cropland converted to settlements (B)		Grassland converted to settlements (C)	
	Sampling area	Total area (land conversion ratio)	Sampling area	Total area (land conversion ratio)	Sampling area	Total area (land conversion ratio)	Sampling area	Total area (land conversion ratio)
Gangwon-do	1606.23	16,804.16 (21.52)	742.33	7766.11 (9.95)	498.24	5212.47 (6.68)	365.67	3825.57 (4.90)
Gyeonggi-do	4859.09	39,459.32 (19.61)	1972.12	16,015.03 (7.96)	1823.63	14,809.21 (7.36)	1063.34	8635.07 (4.29)
Gyeongsangnam-do	1427.73	15,172.30 (14.73)	548.26	5826.32 (5.65)	566.7	6022.25 (5.84)	312.77	3323.72 (3.23)
Gyeongsangbuk-do	2959.98	29,689.50 (25.06)	1084.14	10,874.28 (9.18)	1219.67	12,233.65 (10.33)	656.17	6581.57 (5.55)
Gwangju-si	400.85	3926.65 (24.65)	67.76	663.81 (4.17)	282.92	2771.48 (17.40)	50.16	491.36 (3.08)
Daegu-si	428.97	4381.20 (18.74)	81.70	834.38 (3.57)	249.25	2545.63 (10.89)	98.03	1001.19 (4.28)
Daejeon-si	240.56	2429.99 (15.89)	83.00	838.45 (5.48)	122.6	1238.45 (8.10)	34.95	353.09 (2.31)
Busan-si	509.79	5384.90 (19.41)	106.46	1124.52 (4.05)	248.38	2623.65 (9.45)	154.95	1636.72 (5.90)
Seoul-si	103.18	1017.12 (2.63)	41.15	405.62 (1.05)	51.85	511.12 (1.32)	10.18	100.38 (0.26)
Sejong-si	320.44	3194.43 (42.04)	160.96	1604.55 (21.12)	111.91	1115.58 (14.68)	47.58	474.31 (6.24)
Ulsan-si	510.31	5312.57 (27.14)	216.13	2250.03 (11.49)	207.17	2156.69 (11.02)	87.01	905.85 (4.63)
Incheon-si	249.12	2964.88 (7.94)	76.57	911.32 (2.44)	97.11	1155.73 (3.09)	75.44	897.83 (2.40)
Jeollanam-do	2109.82	23,245.55 (19.13)	423.47	4665.75 (3.84)	1164.00	12,824.73 (10.56)	522.34	5755.07 (4.74)
Jeollabuk-do	1004.15	10,317.00 (12.83)	275.72	2832.82 (3.52)	609.56	6262.86 (7.79)	118.87	1221.32 (1.52)
Chungcheongnam-do	2178.78	22,243.33 (22.70)	735.81	7511.95 (7.67)	935.77	9553.40 (9.75)	507.19	5177.99 (5.28)
Chungcheongbuk-do	916.85	9109.88 (13.39)	677.31	6729.84 (9.89)	46.68	463.83 (0.68)	192.85	1916.21 (2.82)
Jeju-si	810.13	8607.63 (31.52)	343.32	3647.78 (13.36)	273	2900.63 (10.62)	193.81	2059.22 (7.54)
Total	20,635.97	203,260.42 (18.79)	7636.21	74,502.57 (6.89)	8508.44	84,401.37 (7.80)	4491.32	44,356.48 (4.10)

### GHG emission statistics

The GHG inventory was estimated at Tier 1 and App 3 levels using the removal/emission factors specified in the 2006 IPCC GL [8]. The calculated CO_2_ emission was 18.94 MtCO_2_ for the 15-year period from 2005 to 2019, and the annual CO_2_ emission was 1.26 MtCO_2_  yr^−1^ in the same period. At the province level, Gyeonggi-do showed the highest emission (0.27 MtCO_2_ yr^−1^), followed by Gyeongsangbuk-do (0.18 MtCO_2_ yr^−1^), Chungcheongnam-do (0.13 MtCO_2_ yr^−1^), Gangwon-do (0.13 MtCO_2_ yr^−1^), and Chungcheongbuk-do (0.10 MtCO_2_ yr^−1^).

CO_2_ emissions from land converted to settlement (16.39 MtCO_2_ yr^−1^) in South Korea confirmed that emissions from FS had a substantial impact, followed by those from CS (1.45 MtCO_2_ yr^−1^) and GS (1.10 MtCO_2_ yr^−1^) (Table 9). According to KFS Forestry Statistical Yearbook [33, 34], it was confirmed that forests, which were 6.39 Mha in 2005, decreased to 6.33  Mha in 2019. Department of Statistics of South Korea confirmed that the cropland area decreased to 1.82 Mha in 2005 and 1.58 Mha in 2019 [35, 36]. On the other hand, the Cadastral Statistics Annual Report confirmed that the settlement area increased from 0.77 Mha in 2005 to 1.06 Mha in 2019 [18, 37]. Such land conversion affects emissions from settlements, with FS having the most substantial impact as carbon dioxide stocks of trees biomass in forests are estimated to be higher than those in grasslands and croplands, as confirmed.

**Table 9:** Carbon dioxide emission from land converted to settlement in 2005–2019 (Tier 1, Approach 3)

Division	Forest converted to settlements		Cropland converted to settlements		Grassland converted to settlements		Total carbon dioxide emissions (MtCO_2_)	Annual carbon dioxide emissions (MtCO_2_yr^−1^)
	Carbon emissions (MtC)	Carbon dioxide emissions (MtCO_2_)	Carbon emissions (MtC)	Carbon dioxide emissions (MtCO_2_)	Carbon emissions (MtC)	Carbon dioxide emissions (MtCO_2_)		
Gangwon-do	0.47	1.71	0.02	0.09	0.03	0.09	1.89	0.13
Gyeonggi-do	0.96	3.52	0.07	0.26	0.06	0.21	3.99	0.27
Gyeongsangnam-do	0.35	1.28	0.03	0.10	0.02	0.08	1.47	0.10
Gyeongsangbuk-do	0.65	2.39	0.06	0.21	0.04	0.16	2.77	0.18
Gwangju-si	0.04	0.15	0.01	0.05	0.00	0.01	0.21	0.01
Daegu-si	0.05	0.18	0.01	0.04	0.01	0.02	0.25	0.02
Daejeon-si	0.05	0.18	0.01	0.02	0.00	0.01	0.21	0.01
Busan-si	0.07	0.25	0.01	0.05	0.01	0.04	0.33	0.02
Seoul-si	0.02	0.09	0.00	0.01	0.00	0.00	0.10	0.01
Sejong-si	0.10	0.35	0.01	0.02	0.00	0.01	0.38	0.03
Ulsan-si	0.14	0.50	0.01	0.04	0.01	0.02	0.55	0.04
Incheon-si	0.05	0.20	0.01	0.02	0.01	0.02	0.24	0.02
Jeollanam-do	0.28	1.03	0.06	0.22	0.04	0.14	1.39	0.09
Jeollabuk-do	0.17	0.62	0.03	0.11	0.01	0.03	0.76	0.05
Chungcheongnam-do	0.45	1.65	0.04	0.16	0.03	0.13	1.95	0.13
Chungcheongbuk-do	0.40	1.48	0.00	0.01	0.01	0.05	1.54	0.10
Jeju-si	0.22	0.80	0.01	0.05	0.01	0.05	0.90	0.06
Total	4.47	16.39	0.40	1.45	0.30	1.10	18.94	1.26

**Fig. 4:**
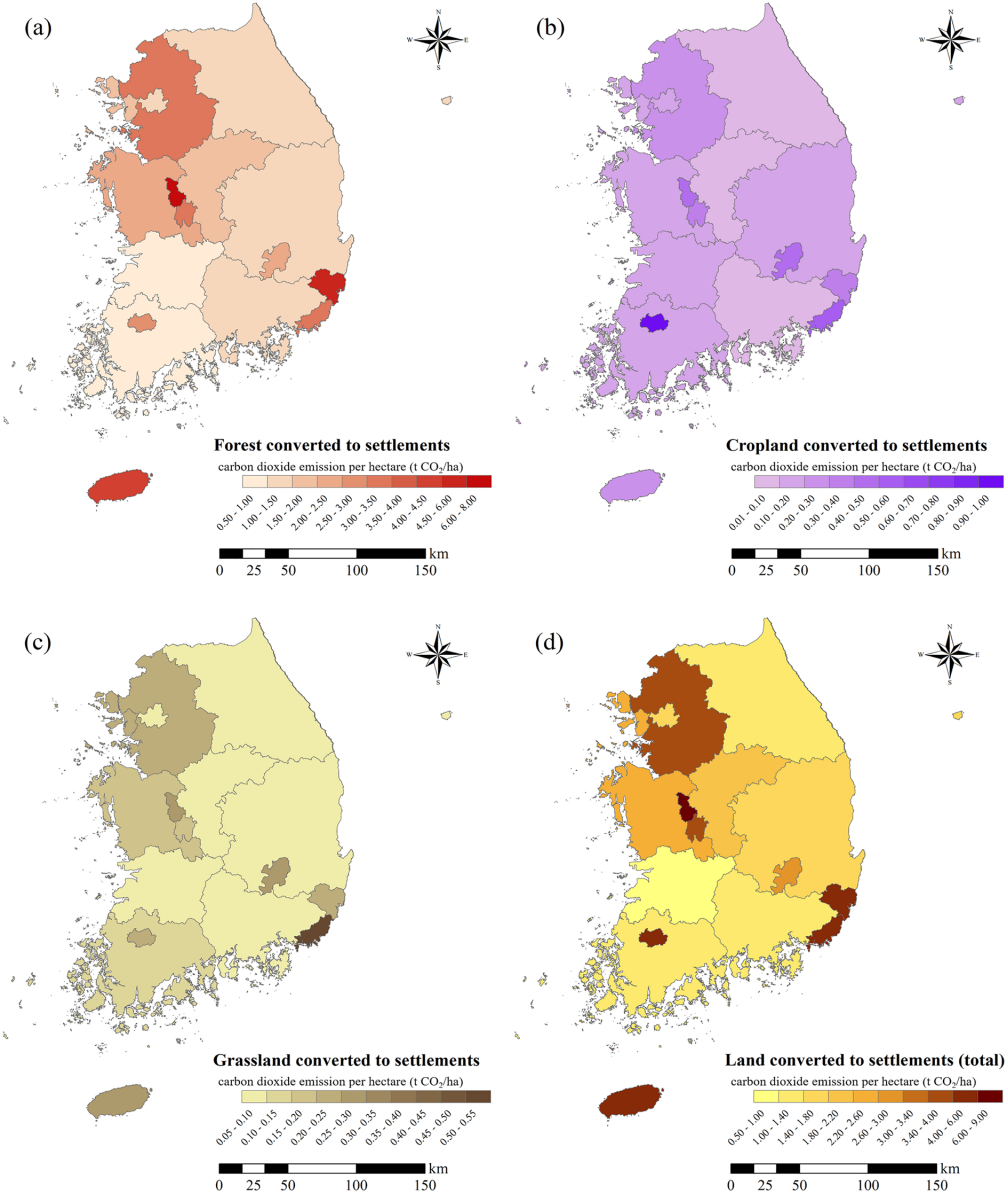
The density of the CO_2_ emission, as represented by ha per CO_2_ from land converted to settlements in 2005–2019. ha per CO_2_ emissions from forest converted to settlements (**a**), cropland converted to settlements (**b**), grassland converted to settlements (**c**), and total ha per CO_2_ emissions (**d**)

The province with the highest CO_2_ emissions from FS was Gyeonggi-do (3.52 MtCO_2_), followed by Gyeongsangbuk-do (2.39 MtCO_2_), Chungcheongnam-do (1.65 MtCO_2_), and Chungcheongbuk-do (1.48 MtCO_2_) (Fig. 4a). For CS, Gyeonggi-do (0.26 MtCO_2_) showed the highest CO_2_ emissions, followed by Jeollanam-do (0.22 MtCO_2_), Gyeongsangbuk-do (0.21 tCO_2_), and Chungcheongnam-do (0.16 MtCO_2_). Finally, CO_2_ emissions in GS were in the order of Gyeonggi-do (0.21 MtCO_2_), Gyeongsangbuk-do (0.16 MtCO_2_), Jeollanam-do (0.22 MtCO_2_), and Chungcheongnam-do (0.16 MtCO_2_). CO_2_ emissions from Gyeonggi-do and Gyeongsangbuk-do were high in all cases. These results appeared in areas with a relatively sizeable region land area, and it was found that this is because the larger the region land area, the larger the area ratio of the permanent settlement area. Therefore, to compare emissions by conversion type, we identified density values that divided total emissions by land area (Table 10, Fig. 4).

**Table 10:** Hectare per carbon dioxide emission from land converted to settlement in 2005–2019

Division	Total land area (ha)	Forest converted to settlements ha per emission (t CO_2_/ha)	Cropland converted to settlements ha per emission (t CO_2_/ha)	Grassland converted to settlements ha per emission (t CO_2_/ha)	Total ha per emission (t CO_2_/ha)	ha per annual emission (t CO_2_/ha∙yr)
Gangwon-do	1,682,967.77	1.02	0.05	0.06	1.12	0.07
Gyeonggi-do	1,019,526.87	3.46	0.25	0.21	3.92	0.26
Gyeongsangnam-do	1,054,055.32	1.22	0.10	0.08	1.39	0.09
Gyeongsangbuk-do	1,903,402.95	1.26	0.11	0.09	1.45	0.10
Gwangju-si	50,112.84	2.91	0.95	0.24	4.11	0.27
Daegu-si	88,348.75	2.08	0.50	0.28	2.85	0.19
Daejeon-si	53,966.35	3.42	0.40	0.16	3.98	0.27
Busan-si	77,007.48	3.21	0.59	0.53	4.33	0.29
Seoul-si	60,522.86	1.47	0.15	0.04	1.66	0.11
Sejong-si	46,491.24	7.59	0.41	0.25	8.26	0.55
Ulsan-si	106,208.62	4.66	0.35	0.21	5.22	0.35
Incheon-si	106,522.59	1.88	0.19	0.21	2.28	0.15
Jeollanam-do	1,234,809.27	0.83	0.18	0.12	1.13	0.08
Jeollabuk-do	806,983.58	0.77	0.13	0.04	0.94	0.06
Chungcheongnam-do	824,616.83	2.00	0.20	0.16	2.36	0.16
Chungcheongbuk-do	740,695.49	2.00	0.01	0.06	2.07	0.14
Jeju-si	185,021.13	4.34	0.27	0.28	4.88	0.33
Total	10,041,259.93	1.63	0.14	0.11	1.89	0.13

In the case of FS, Sejong-si showed the highest emissions at 7.59 tCO_2_/ha, followed by Ulsan-si (4.66  tCO_2_/ha) and Jeju-si (4.34 tCO_2_/ha). Similar to these results, Kim et al. [38] confirmed the land conversion area between 2009 and 2015 according to sub-classified land cover in Sejong-si and found that the conversion area from forest to urbanized area was the largest at 3.91 km^2^ was confirmed. It seems that the emissions of FS appeared to be the largest due to this. In the CS, Gwangju-si showed the highest emission at 0.95 tCO_2_/ha, followed by Busan-si (0.59 tCO_2_/ha) and Sejong-si (0.41 tCO_2_/ha). GS was the highest in Busan-si with 0.53 tCO_2_/ha, followed by Jeju-si (0.28 tCO_2_/ha) and Sejong-si (0.25 tCO_2_/ha). The results by land conversion type confirmed that CO_2_ emissions per hectare are substantial in particular metropolitan cities where the development pressure is high and the land area is relatively small.

### Verification

Uncertainty assessment for the construction of activity data and the development of emission and removals factors is a requirement of the GHG inventory. However, South Korea currently does not validate the LULUCF sector’s uncertainty except for the forest’s emission and removals absorption factors [39], and the data for validation are insufficient. There is a limit to estimating the uncertainty of activity data, as there is no statistical data on the conversion area and associated biomass from South Korea [40, 41]. Therefore, recognizing that there is a limit to the detailed verification of the results of this study, we calculated the GHG emissions using the land converted to settlements area reported by the MOLIT. The report identified and presented the settlement area converted from other land categories using cadastral maps as of 2005 and 2018 (Table 11) [42]. As a result of estimating CO_2_ emissions using these data, it was confirmed that the emissions from FS were 14.58 MtCO_2_, and the results of this study were 16.39 MtCO_2_. In the case of CS, 1.56 MtCO_2_ was confirmed, which was more substantial than 1.45 MtCO_2_ in this study. Finally, in the case of GS, 0.01 MtCO_2_ was smaller than the result of this study (1.10 MtCO_2_).

**Table 11:** The area of settlements converted from other lands surveyed from literature and the result CO_2_ emissions

Division	Area (ha)	Carbon dioxide emission (MtCO_2_)	Annual carbon dioxide emission (MtCO_2_/yr)
Forest converted to settlements	66,261	14.58	0.97
Cropland converted to settlements	90,467	1.56	0.10
Grassland converted to settlements	1418	0.01	0.00
Total	192,230	16.15	1.08

## Discussion

Boundary divisions within the LULUCF sector can appear in various forms because they are affected by various biophysical phenomena. Therefore, stratification of land is required according to variables such as vegetation, climate zone, soil type, management type, intensity, and disturbances [43]. In countries such as South Korea, where GHG inventory estimated only by land area statistics is calculated due to insufficient spatial data or multiple different data sources for each land-use category, consistent standards should be established by identifying the characteristics of the land-use category.

Although the cadastral map and land cover map can be used for App 3 in South Korea, we only used the cadastral map for three reasons. First, the urban area in the land cover map covers only the impermeable layer in the city, which does not include green areas. Thus, neither GHG emissions nor removals from the urban area can be included in the GHG inventory using this map. However, the IPCC defined settlements to contain soils or vegetation in urban areas. Second, although the GHG inventory for managed forest land needs to be estimated under the Kyoto Protocol, it is difficult to reflect this management status in the land cover map. The cadastral map, which is based on parcel units, can be easily applied in this scenario. Finally, the definitions of forest land and cropland based on the IPCC differ from those in the land cover map in South Korea. For this reason, this study proposed a method of classifying land-use types using the cadastral map for land not currently spatially divided and constructing a LUC matrix accordingly.

In contrast, many countries conduct land-use classification using the land cover map using information obtained from remote sensing. Land cover classes are measurable physiognomic characteristics, such as vegetation height, tree crown, or biomass density within the spatial unit. Thus, the land cover concept is not strictly distinguished from the land use concept. However, land use is characterized by the arrangements, activities, and inputs of people [19]. Therefore, it is necessary to investigate the relationship between land use and land cover to utilize the land cover map, and spatial thresholds or ranges must be set. However, as in this study, when land use boundaries are set using a cadastral map (parcel unit), it is typically necessary to construct activity data for GHG removal/emissions sources, particularly the tree biomass, compared with a land cover map [44, 45]. This method is more labor- and cost-intensive than using land cover methods. Therefore, to maintain the consistency of the land-use category, a LUC matrix should be constructed by selecting an available method for the national situation.

When using several spatial data specific to each category, such as a digital forest type map and smart farm map in South Korea, spatial overlap and missing areas must be checked, and post-processing is required. In the United States, a LUC matrix is constructed through hierarchical division to minimize spatial data overlap by land use category [9]. However, in South Korea, because several ministries are involved in estimating the GHG inventory in the LULUCF sector, it is necessary for ministries to agree on land-use boundaries for this hierarchical division. A missing area can be supplemented by utilizing remote-sensing data and consistent standards. Remote sensing data has been widely used in other Annex I countries to more accurately classify the LULUCF sector [23, 46–48].

When constructing a LUC matrix, many Annex I countries such as Italy, the United States, and Sweden use fixed sample areas and sample points to analyze the LUC and construct activity data [9, 13, 49]. Assessing the precision and accuracy of large-area surveys, such as in the wall-to-wall method, is complex and involves many survey populations; thus, a considerable amount of information is required to plan and implement the survey, data analysis, and estimation [36]. However, precise spatial analysis of the wall-to-wall method is required to analyze the LUC more accurately. In this study, the sampling and wall-to-wall methods were combined, enabling analysis of the LUC in a more accurate, consistent, and cost-effective manner.

In GHG inventory statistics, the results for GHG emissions tend to be overestimated, and Tier 1 default factors do not precisely represent the situation in South Korea. The Tier 2a default factors for tree biomass are presented in the IPCC GL but differ from the experimental results obtained in South Korea [50–54]. Therefore, it is necessary to develop GHG removal/emission factors suitable for the national situation to estimate the GHG inventory more accurately. In addition, in this study, verification value differences occurred from the difference between land classification according to the purpose of land use defined by the cadastral map and the actual land-use status. The forests of the cadastral map in South Korea are defined as containing bamboo, rock, gravel, sand, and wetland that form forests. Accordingly, there is a difference in the area of forest land and where actual trees and shrublands exist. Therefore, it does not include the urban forest. Cropland is defined as including the field, paddy field, and orchard of the cadastral map, and the land within these areas includes land for housing, which may differ from the areas for actual cropland. Furthermore, there is an area mismatch due to illegal cultivation in the forest, in the case of grassland defined as including ranch of the cadastral map. The problem is that grassland in the forest and grassland in the residential area is not included [41]. Therefore, to solve these problems and improve GHG inventory estimation, it is necessary to continuously understand the division of the purpose of land use and the actual status of land use in South Korea by continuously constructing land conversion data and activity data. Finally, for reliable GHG inventory estimates, developing an uncertainty assessment methodology for the LULUCF sector for the Korean situation is necessary as conducting assessments accordingly.

South Korea established a 2nd National GHG statistics management plan, including LUC matrix construction in the LULUCF sector, the development of activity data, and GHG removal/emission factors by land-use category in preparation for the Global stock take under Article 13 of the Paris Agreement. Our results will contribute to the accurate and consistent analysis of the GHG inventory in the LULUCF sector. Furthermore, in a country where it is difficult to construct a LUC in the same situation as in South Korea, a system and data for constructing a LUC matrix can be prepared based on our methodologies and results.

## Conclusion

To improve the approach level used by the LULUCF sector in South Korea, which is currently reported at the level of Approach 1, we reviewed available spatial data and constructed a LUC matrix. Through this method, it was possible to estimate the GHG inventory that can identify LUC. In addition, this study conducted a detailed discussion on materials and methods for constructing the LUC matrix and proposed a methodology to improve the approach level. Through this process, it is possible to provide detailed information on LUC in South Korea’s settlements category and a LUC matrix construction methodology for countries with similar problems to South Korea. In addition, it was confirmed that data accumulation and verification methodologies for LUC was needed to increase the reliability of the LULUCF sector's GHG inventory.

## Data Availability

Cadastral map is available online through the National Spatial Infrastructure Portal of the South Korea website (http://data.nsdi.go.kr/dataset). Ortho-images were obtained from the National Geographic Information Institute (https://www.ngii.go.kr/kor/). Forest aerial photographs were obtained from the Forest Geospatial Information System (https://fgis.forest.go.kr/).
